# Incentives can spur COVID-19 vaccination uptake

**DOI:** 10.1073/pnas.2109543118

**Published:** 2021-08-19

**Authors:** Heike Klüver, Felix Hartmann, Macartan Humphreys, Ferdinand Geissler, Johannes Giesecke

**Affiliations:** ^a^Department of Social Science, Humboldt University of Berlin, 10117 Berlin, Germany;; ^b^Institutions and Political Inequality Research Unit, WZB Berlin Social Science Center, 10785 Berlin, Germany;; ^c^Department of Political Science, Columbia University, New York, NY 10027

**Keywords:** COVID-19, vaccination, incentives, herd immunity, hesitancy

## Abstract

Recent evidence suggests that vaccination hesitancy is too high in many countries to sustainably contain COVID-19. Using a factorial survey experiment administered to 20,500 online respondents in Germany, we assess the effectiveness of three strategies to increase vaccine uptake, namely, providing freedoms, financial remuneration, and vaccination at local doctors. Our results suggest that all three strategies can increase vaccination uptake on the order of two to three percentage points (PP) overall and five PP among the undecided. The combined effects could be as high as 13 PP for this group. The returns from different strategies vary across age groups, however, with older cohorts more responsive to local access and younger cohorts most responsive to enhanced freedoms for vaccinated citizens.

Vaccination is the most important instrument to sustainably contain the COVID-19 pandemic. However, in order to stop the pandemic, it is estimated that at least 70 to 85% of the population needs to be vaccinated (1). Recent survey evidence suggests that this threshold cannot be met in many countries (2). Accordingly, the vaccine rollout in a number of countries has shown that it is comparatively easy to vaccinate the first half of the population. However, getting the undecided and more-hesitant citizens vaccinated is a real challenge. Decision makers therefore debate which strategies can increase vaccination uptake. In this study, we contribute to this discussion by evaluating the effectiveness of three strategies that affect citizens’ incentives to vaccinate.

While vaccination hesitancy is a major challenge in the fight against the COVID-19 pandemic, there is still limited research on this topic. Early experimental studies conducted in a purely hypothetical setting before vaccines were available arrived at mixed results (3). Recent studies conducted after the first vaccines were approved have found that basic information about the vaccine and priming social approval benefits can increase vaccination uptake (4). However, there is no evidence so far on how policy instruments that go beyond information campaigns and framing can reduce vaccination hesitancy.

We address this gap and test the effect of three strategies that governments can apply to raise the willingness to get vaccinated against COVID-19: granting freedoms, financial remuneration, and vaccination at local doctors. We identified these strategies based on previous research and the current political debate revolving around the rollout of the COVID-19 vaccines. The first strategy, granting freedoms, refers to policies that only reinstall certain liberties to people who are vaccinated, while penalizing those without a vaccination (5). The second strategy, financial remuneration, refers to providing citizens monetary incentives for vaccination uptake (6, 7). The third strategy, vaccination at local doctors, rests on two ideas, namely, the reduction of transaction costs and increasing trust (8). Prior research has demonstrated that transaction costs are a major reason why citizens do not uptake services (9), while research on vaccination hesitancy has shown that trust in institutions is a major predictor for vaccination uptake (10).

## Results

To evaluate the effectiveness of these strategies, an experimental study embedded in a nationally representative survey was fielded in Germany. We recruited 20,500 respondents from 5 March to 25 March 2021 (for details, see *SI Appendix*). Among our sample, we found that 7% of respondents were vaccinated, and 60% would accept a vaccine. Another 17% remained undecided, and 16% would refuse to get vaccinated. Germany takes a middle position in vaccine hesitancy across countries—ref. 2 finds an average acceptance rate around 72% across 19 countries, although with wide variation. As in other countries examined in ref. 2, institutional trust is an important correlate of hesitancy in Germany. Moreover, the arguments given for vaccine hesitancy are not specific to the German context: When respondents in our sample give an account for their hesitancy, about two-thirds describe concerns over the side effects, or adverse long-term effects of the vaccine, with fewer (19%) discounting the seriousness of corona. These features give some confidence that findings from Germany have implications that extend beyond the case.

In our experiment, participants were randomly exposed to vignettes about a hypothetical policy context that varied along three dimensions (see *SI Appendix*): freedoms for vaccinated people (yes, no), financial incentives for vaccination (none, 25 euros, 50 euros), and vaccination at local doctors (yes, no). Respondents were asked about their willingness to get vaccinated under these different policy scenarios. Even though these are only hypothetical policy scenarios, previous research has shown that the same factors that drive hypothetical choices in survey experiments can predict comparable choices in the real world (11). To further check whether reported intentions are correlated with real-world behavior, we compared the vaccination status of survey participants in a second survey wave conducted 2 mo later and found high correspondence (see *SI Appendix*, section G).

**Fig. 1.**
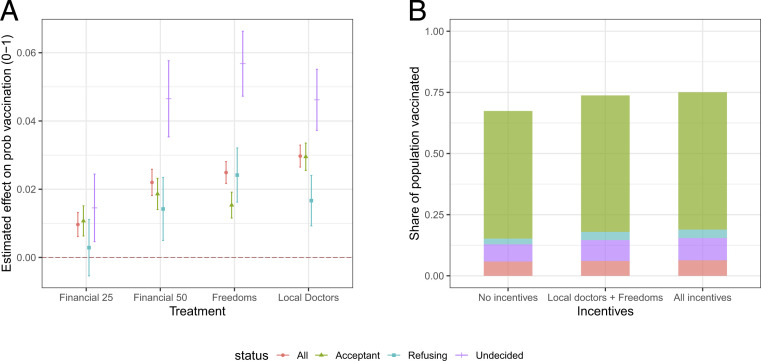
(*A*) Effects of mass vaccination scenario attributes on the probability that respondents take the vaccine in the scenario. Dots with vertical lines indicate point estimates with robust 95% CIs from least squares regression, accounting for individual-level fixed effects. (*B*) Predicted shares of population that would be vaccinated under different incentives.

Fig. 1*A* plots the estimated average effects of the three policy strategies on reported uptake. Each treatment estimate should be interpreted relative to a control vignette that differs on the factor in question. The outcome is measured on a zero-to-one scale and can be interpreted as a self-report of a respondent’s probability of accepting COVID-19 vaccination.

Three out of four treatments have sizable and statistically significant effects on the reported willingness to get vaccinated, with estimated effects ranging between one and three percentage points (PP). We observe the lowest treatment effect for the low financial incentive (25 euros) with 1 PP, 2.2 PP for high financial incentives (50 euros), a 2.5-PP increase for the additional freedoms, and a 3-PP increase for vaccinations at the local doctor (all significant at p<0.001). Comparing effect sizes, we find that doubling the financial incentive corresponds to a more than doubling of the effect on vaccination uptake.

Interpreting the implications of the effect sizes for overall vaccination levels depends on the sizes of the different groups that respond differently to these interventions. As shown in Fig. 1*B*, applying incentives is associated with gains from about two-thirds vaccinated to three-quarters vaccinated. A large share of these gains is achieved from incentives recently implemented, or likely to be implemented, in Germany: local doctors and enhanced freedoms.

### Treatment Effects for the Hesitant.

Subsetting by prior hesitancy, we see that the largest effects are for undecided respondents. Respondents who refuse to get vaccinated are overall less likely to respond to any treatment conditions. However, for those who remain undecided, the high financial treatment increases by five PP the share of respondents who say that they will get vaccinated (p<0.001). Similarly, the possibility to get vaccinated at the local doctor increases the share by five PP (p<0.001). We observe the strongest effect (six PP) among the undecided for the personal freedoms treatment. The treatment effect is statistically different from the high financial and the local doctor treatment at p<0.05.

Using an omnibus test, we compare the combined effects of all treatments (freedoms, local doctors, 50-euro financial incentives) against a combined control group (no freedoms, no local doctors, no financial incentives). Among the undecided, we see a 13-PP increase in acceptance from a baseline of 40% (p<0.001) (see *SI Appendix*). Given that vaccination acceptance is just a few percentage points below the estimated threshold for herd immunity in many countries (2), these are sizable effects that could help to achieve community immunity.

### Heterogenous Effects.

We supplement this analysis with a preregistered machine learning approach that provides insights regarding which strategies are most effective for which population subgroups. We use a causal forests approach (12) which is a specific application of the generalized random forests algorithm (13). We estimate conditional average treatment effects across all covariates in our dataset (see *SI Appendix*).

**Fig. 2.**
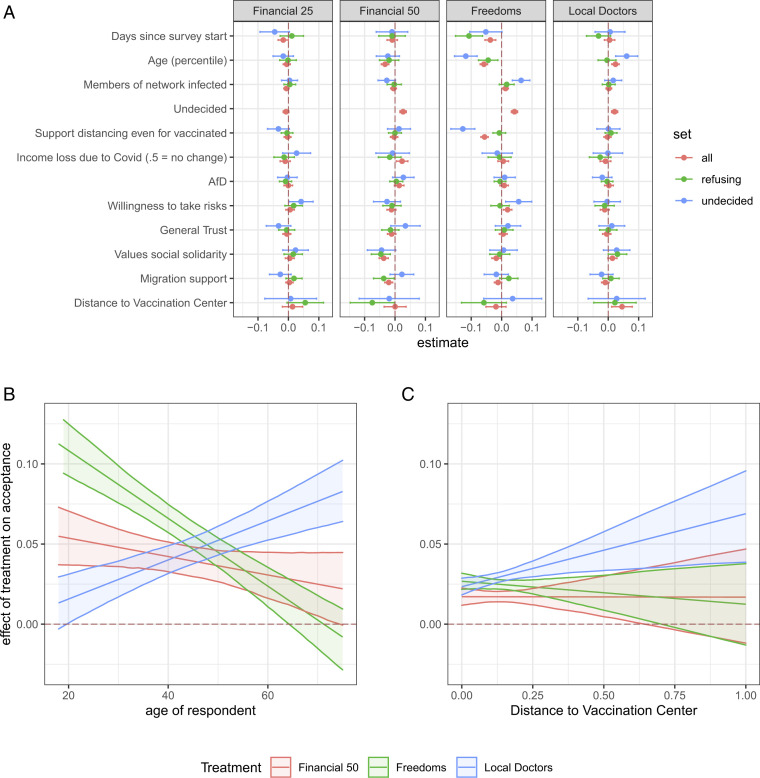
Heterogeneous treatment effects. (*A*) Features that account for heterogeneity in treatment effects. Dots indicate the coefficient for the best linear projections of covariates on effect heterogeneity, with positive (negative) numbers indicating that average effects are more positive at higher (lower) values of the covariate. The 95% CIs are indicated with horizontal lines. (*B*) Treatment effects depending on the age of respondents. Shown are heterogeneous treatment effects for each factor for undecided citizens. (*C*) Treatment effects depending on the distance to the closest vaccination center. Distances are calculated as minimum between centroid of respondent zip code and closest vaccination center. Source for distance is: https://overpass-turbo.eu/s/10TL, downloaded 29 July 2021.

The most important substantive finding is that the effectiveness of the strategies varies by age group (Fig. 2*B*). Older cohorts are more responsive to local access, and younger cohorts are most responsive to enhanced freedoms. In addition, we find that respondents who stated that they were undecided about getting vaccinated showed higher treatment effects for high financial incentives and personal freedoms. Fig. 2*B* depicts the treatment effects depending on age for undecided respondents. Older cohorts are more responsive to the opportunity to get vaccinated at local doctors, while younger cohorts are most receptive to granting freedoms to vaccinated citizens. Finally, Fig. 2*C* shows that the estimated effect of vaccinations at the local doctor considerably increases with the distance of respondents to vaccination centers. This finding suggests that the reduction of transaction costs is a likely mechanism underlying the effect of the local doctor treatment.

A drawback of survey experiments is that responses may be affected by social desirability. Three points, however, speak against social desirability accounting for our findings. First, if social desirability were driving results, we should observe similar effects for the different treatments. We, however, find that a monetary incentive of 25 euros has a much smaller effect on uptake than all the other incentives. Second, we do not see an increase in overall acceptance between rounds (we see a small decline), and, moreover, estimated effects for the first and second round of the experiment, analyzed separately, are largely identical. These results speak against the idea that respondents adapted to become more supportive of vaccination or more reactive to proposed treatments. Third, and most important, reported vaccination willingness is strongly correlated with subsequent real-world behavior. We show this by comparing reported vaccination intention with the vaccination status of respondents 2 mo later (see *SI Appendix*, section G).

## Conclusion

Our results suggest that governments can increase vaccine uptake through three different policy instruments, namely, providing freedoms, financial remuneration, and vaccination at local doctors. Granting liberties that are not available to nonvaccinated citizens can encourage uptake, especially among the undecided. Similarly, financial rewards can also increase uptake, but payments have to be sizable. Finally, vaccination at local doctors is an effective instrument, most likely because it reduces transaction costs for citizens. These strategies can be combined and largely appear not to substitute for each other.

While all three strategies have positive average effects, our findings suggest that the scope for altering behavior using incentives like those we study among respondents that are refusing vaccination is limited. Governments can do better by focusing on undecided citizens, for whom combined effects could be as high as 13 PP. In addition, governments seeking to increase vaccination uptake among undecided younger cohorts may see greater returns from enhancing freedoms, while governments focused on undecided older citizens will see greater returns from ensuring provision at local doctors. Vaccination at local doctors and a vaccination passport have, in the meantime, been implemented in Germany. Our results suggest that these were likely good strategies for the German government to have adopted. The additional gains from payments are now likely relatively small in the German case. More generally, the results suggest that all three strategies may be effective in countries in which vaccine rollout is in earlier stages.

## Methods and Materials

### Consent and Ethics.

The study was preregistered before data collection on 4 March 2021 at https://doi.org/10.17605/OSF.IO/H8RKB. Ethical approval was obtained from the institutional review board at Humboldt-Universität zu Berlin (HU-KSBF-EK_2021_0003). On recruitment, participants were informed about the content of the study and the handling of the data. They were provided with a consent form and were only directed to the survey if they provided consent and chose to take part in the study.

## Supplementary Material

Supplementary File

## Data Availability

Replication data for the complete analysis are provided on Github (https://wzb-ipi.github.io/covid_hesitancy_2021) (14). A copy of the questionnaire used in this study can be obtained on Open Science Framework (OSF) (https://doi.org/10.17605/OSF.IO/H8RKB). Regression tables and extended analyses are also on OSF (https://doi.org/10.31219/osf.io/ax6pw).
