# Supplementary material for: Incentives can spur COVID-19 vaccination uptake
Source: Proc Natl Acad Sci U S A. 2021 Aug 19;118(36):e2109543118. doi: 10.1073/pnas.2109543118 (PMC8433545; doi:10.1073/pnas.2109543118)
Supplement: Supplementary File [file pnas.2109543118.sapp.pdf]

Incentives can spur Covid-19 vaccination uptake

Heike Klüver, Humboldt-Universität zu Berlin
Felix Hartmann, Humboldt-Universität zu Berlin
Macartan Humphreys, WZB Berlin Social Science Center/Columbia University
Ferdinand Geissler, Humboldt-Universität zu Berlin
Johannes Giesecke, Humboldt-Universität zu Berlin

Supporting Information (SI) Appendix

Contents

A Sample & Recruitment **2**

B Survey questionnaire **2**

C Experimental Design **3**

D Correlates of vaccine hesitancy **5**

E Omnibus treatment **5**

F Heterogeneous Treatment Effects **5**

G Correspondence with real-world behaviour **7**

A Sample & Recruitment

Our population of interest consists of all German citizens aged 18 to 75 years. We rely on a representative sample of 20,500 citizens across Germany. In order to conduct the factorial survey experiment, we fielded the experiment relying on the online access panel of the survey company Respondi. Respondi relies on online channels and offline channels to recruit new panelists for its online panel. After completing a profiling questionnaire covering basic sociodemographic information, panelists are then invited to participate in surveys. Respondi compensates its panelists for completing a survey. In our study, the incentive was EUR 0.75 for a median length of interview (LOI) of approximately 15 minutes.

B Survey questionnaire

We first asked the respondents about their willingness to get vaccinated prior to the treatment in order to be able to estimate heterogeneous effects for different subgroups of the population, namely those who are more acceptant, those who refuse to get vaccinated, and those who are undecided. Including this pre-treatment question makes it possible to assess for which of these subgroups the three strategies work best. To avoid priming or consistency biases, we tried to maximize the gap between asking the pre-treatment question about the willingness to get vaccinated and the administration of the survey experiment. More specifically, participants were asked right at the beginning of the questionnaire whether they are already vaccinated (Yes / No) and if not, whether they plan on getting vaccinated (Yes / No / Have not decided yet). A number of other questions on other Corona-related issues as well as on general political attitudes and political behaviour followed so that the attention of respondents was directed to other topics before exposing them to the vignette. Between the first pre-treatment question on vaccination willingness and the survey experiments, participants responded to 25 different questions. For the factorial survey experiment participants were randomly introduced to two out of twelve possible hypothetical scenarios of the three policy instruments (special rights for vaccinated people, financial incentives for vaccination (25 or 50 Euro) and vaccination at local medical practices). Subsequently, they were asked to indicate their willingness to get vaccinated on a scale from 0 to 10. We note that although those who are willing to get vaccinated in the first place report a much higher willingness to get vaccinated under all hypothetical scenarios, we nevertheless observe variation for this group in their responses to this question. For this reason it is possible to find positive treatment effects even among the "acceptant" group. The exact question wording and the order of the survey questions can be found here: https://osf.io/h8rkb/.

We implemented two further analyses to check for potential priming effects. First, we ran separate analyses for the first and second round of the experiment. We find that the effects do not vary very much between the first and second iteration, which suggests that priming does not play a major role. Second, we tested whether the treatment during the first round affects responses in the second round of the experiment. By and large, we do not find that the first experimental treatment has an effect on the vaccination probability in round two. Only among acceptant respondents, we find that respondents who were exposed to the local doctors or freedoms treatment in the first round, were more likely to say yes in the second round.

C Experimental Design

In the factorial survey experiment, participants were randomly exposed to different hypothetical policy instruments (special rights for vaccinated people, financial incentives for vaccination and vaccination at local medical practices). Subsequently, we questioned respondents about their willingness to get vaccinated under these different policy scenarios. The experiment relies on a single profile forced choice conjoint experiment using 2x2x3 factorial design, with factors assigned with independent probabilities. Each respondent was asked to indicate the willingness to get vaccinated under two hypothetical policy contexts, and for each profile we randomly assigned the values of all attributes. However, respondents did not see that same profile twice. The treatment consists of a vignette consisting of the following policy factors Z with randomly assigned levels.

- **Treatment Factor Z:** Freedoms
  - Control (0): There are **no special regulations** for vaccinated people even when the Corona incidence is high. For example, they cannot travel again, visit cinemas, restaurants or concerts and are still subject to contact restrictions.
  - Treatment (1): **Special regulations** apply to vaccinated people. For example, even when the Corona incidence is high, they can travel again, visit cinemas, restaurants or concerts and are not subject to any contact restrictions.
- **Treatment Factor Z:** Local Doctors
  - Control (0): Eligible citizens can get vaccinated against Corona at the nearest vaccination center **but not at their local doctor**.
  - Treatment (1): Eligible citizens get vaccinated against Corona at the nearest vaccination center **or at their local doctor**
- **Treatment Factor Z:** Financial Incentives

- Control (0): Citizens who are vaccinated **will not receive any allowance** after receiving the vaccination.
- Treatment 1 (1): Citizens who get vaccinated **receive an expense allowance of 25 Euros** after receiving the vaccination.
- Treatment 2 (2): Citizens who get vaccinated **receive an expense allowance of 50 Euros** after receiving the vaccination.

Our main outcome variable is the vaccination probability which takes on values between 0 and 10. We rescale the outcome to take on value between 0 and 1.

- **Outcome: Vaccination Probability** Please use this scale to indicate how likely it is that you would be vaccinated against Corona under these conditions.
  - 0 I will definitely not be vaccinated against Corona
  - 1
  - 2
  - 3
  - 4
  - 5
  - 6
  - 7
  - 8
  - 9
  - 10 I am sure to get vaccinated against Corona

Main Estimation. We seek to estimate the average effect of each treatment, averaged over other treatment conditions. We estimate treatment effects using an OLS regression with individual level fixed effects and heteroskedasticity-robust standard errors:

$$\begin{aligned} Y_{it} \quad = \quad & \beta_0 + \beta_1 Z^1_{it} + \beta_2 Z^2_{it} + \beta_3 Z^{3,\text{ low}}_{it} + \beta_4 Z^{3,\text{ high}}_{it} + \\ & \beta_5 Z^1_{it} Z^2_{it} + \beta_6 Z^1_{it} Z^{3,\text{ low}}_{it} + \beta_7 Z^1_{it} Z^{3,\text{ high}}_{it} + \\ & \beta_8 Z^2_{it} Z^{3,\text{ low}}_{it} + \beta_9 Z^2_{it} Z^{3,\text{ high}}_{it} + \beta_{10} Z^1_{it} Z^2_{it} Z^{3,\text{ low}}_{it} + \\ & \beta_{11} Z^1_{it} Z^2_{it} Z^{3,\text{ low}}_{it} + u_i + \epsilon_{it} \end{aligned}$$

where Y is a continuous variable measuring the reported likelihood of vaccination of participant i for two policies ($t \in \{1,2\}$) each described by three conditions Z^1 (Freedoms), Z^2 (Local Doctor), $Z^{3,low}$ (Financial remuneration 25 Euros), $Z^{3,high}$ (Financial remuneration 50 Euros)). Conditions are centered with 0 mean. With this normalization β_1 to β_4 capture the average fixed effects averaged over conditions of other variables.

D Correlates of vaccine hesitancy

Vaccine hesitancy in Germany, our data show, strongly reflects political and social divisions. Younger respondents and women are more likely to be undecided, but these relations are not substantively large. Similarly employment status, and professional position do little to explain hesitancy in Germany, though education is strongly (negatively) correlated with undecidedness and vaccine refusal. A constellation of measures of general trust, trust in institutions, and support for the populist right AfD political party powerfully explain refusal. A majority of the refusing respondents either support the AfD (20%) or support no party (37%). Among those accepting, there is just 3% support for the AfD and 21% support no party.

E Omnibus treatment

The omnibus analysis compares responses to policies with no incentives to policies with 50 Euro financial incentives, plus enhanced freedoms, plus reduced transactions costs. Most respondents saw only one of these two conditions and so fixed effects are not employed in this analysis. Instead standard errors are clustered at the individual level to account for cases in which a subject gave responses for both conditions.

F Heterogeneous Treatment Effects

In the second step of the analysis we use the Generalized random forests (grf) package (CRAN, 0.10.2) to identify variation in treatment effects. The approach randomly partitions the data and identifies which regression trees predict the largest variation in the magnitude of effects. The algorithm selects variables and determines whether to "split" a variable in order to maximize a heterogeneity criterion. Repeated application produces a "tree" with leafs at the end of the tree containing observations that split in the same way at all decision points. The results provide a rough diagnostic of variable importance: "simple weighted sum of how many times feature i was split on at each depth in the forest." Fig. 2 A displays the most common predictors across treatments. It is possible that a variable is predictive of heterogeneity, but is accorded low importance if it is highly correlated with other predictive

variables .

Predicted effects are generated by a weighted average of outcomes for each observation's neighbors—where neighbors are observations that more commonly end up in the same leaf as an observation. We also pre-registered to examine heterogeneous treatment effects across self-reported partisanship, political ideology, risk aversion, and trust. However, we find no strong evidence of heterogeneity across those variables.

In order to shed more light on whether increased trust or reduced transaction costs account for the effect of the local doctors treatment, we conducted an additional analysis in which we compiled data on the location of vaccination centers in Germany and calculated the distance between the place of residence of respondents (measured by the zip code that they indicated in the survey) and the location of the closest vaccination center. Data on the location of vaccination centers was obtained from: https://overpass-turbo.eu/s/10TL via https://wiki.openstreetmap.org/wiki/Impfzentren_in_Deutschland."

Pre-Treatment Covariates We collect a set of pre-treatment covariates. All of those variables are include in the analysis of the heterogenous treatment effects. Fig. 2 A shows the most common predictors across treatments.

- Survey date, Days since survey start, Previous Covid surveys
- Prior on freedoms, Prior on payments, Prior on transaction costs
- Male respondent, Gender, Age, Age (percentile)
- Non German Citizen, Born outside of Germany, Migration background of parents
- Postcode, State, In former East Germany
- Household size, Household size 3 or more
- In full time employment, Employed, Professional position
- Education, Years of Education, Household income
- Previously had Covid, Members of network infected, Good general health, Covid pre-existing conditions
- Respondent has been vaccinated, Number of Vaccination(s), Share of network vaccinated
- Vaccination eligibility, Vaccination invitation, Vaccination intent, Refusals, Undecided, Acceptant
- Seeks Covid information, Believes media exaggerates risk

- Compliance: mask rules, Compliance: distance rule, upport distancing even for vaccinated, Income loss due to Covid (.5 = no change)
- Political interest, Voted last elections, Voting decision last elections, Right leaning, AfD, Conservatives, Liberals, Green Party, Left Party, Social Democrats, No party identification, Party id
- Willingness to take risks, Fear of long term effects, Fear admission process, Fear effectiveness, Fear side effects, Covid not dangerous
- General Trust, Trust state government, Trust experts, Trust federal government, Trust media, Trust healthcare
- Values social solidarity, Values international solidarity, EU support, Migration support

G Correspondence with real-world behaviour

To further check whether reported attitudes in our survey are correlated with real-world behaviour, we analyzed data from a subsequent second survey wave which we fielded among the respondents of the first wave two months later (N=13,782; Field time: 29 April-10 May 2021). We find that the reported (post-treatment) likelihood of getting vaccinated under the two hypothetical scenarios in wave 1 (based on reported post-treatment vaccination likelihood) is strongly related to the reported vaccination status of participants in wave 2. Among those who reported the highest likelihood of getting vaccinated under the presented hypothetical scenario in wave one, 47.7 % were already vaccinated at wave two in April/May (at a time when not everyone was eligible for vaccination). By contrast, only 5.2% of the respondents who reported the lowest likelihood of getting vaccinated under the presented hypothetical scenarios were vaccinated at wave two. We additionally checked how the reported pre-treatment willingness to get vaccinated in the first survey wave in March is related to actual vaccination status two months later. The share of vaccinated citizens is highest among those participants who reported that they would get vaccinated (39.0%) and it is lowest in the group of respondents who refused to get vaccinated (6.3%) in March while 12.9% of the undecided got vaccinated.
